# Illustrating Instrumental Variable Regressions Using the Career Adaptability – Job Satisfaction Relationship

**DOI:** 10.3389/fpsyg.2019.01481

**Published:** 2019-06-28

**Authors:** Grégoire Bollmann, Serguei Rouzinov, André Berchtold, Jérôme Rossier

**Affiliations:** ^1^Department of Psychology, University of Zurich, Zurich, Switzerland; ^2^NCCR LIVES, University of Lausanne, Lausanne, Switzerland; ^3^Institute of Social Sciences, University of Lausanne, Lausanne, Switzerland; ^4^Institute of Psychology, University of Lausanne, Lausanne, Switzerland

**Keywords:** affect, causal inference, instrumental variable regressions, career adaptability, job satisfaction, personality

## Abstract

This article illustrates instrumental variable (IV) estimation by examining an unexpected finding of the research on career adaptability and job satisfaction. Theoretical and empirical arguments suggest that in the general population, people’s abilities to adapt their careers are beneficial to their job satisfaction. However, a recent meta-analysis unexpectedly found no effect when personality traits are controlled for. We argue that a reverse effect of job satisfaction on career adaptability, originating from affective tendencies tied to personality, might explain this null effect. Our argument implies that the estimates obtained with traditional ordinary least squares (OLS) regressions are biased by endogeneity, a correlation between an explanatory variable and the error term in a regression model. When experimental manipulations are impossible, IV estimations, such as two-stage least squares (2SLS) regressions, are one possible solution to the endogeneity problem. Analyzing three waves of data from a sample of 836 adults, the concurrent and time-lagged effect of job satisfaction on career adaptability was revealed to be more consistent than the reverse. Our results provide an explanation, rooted in affective dispositions, as to why recent meta-analytical estimates unexpectedly found that career adaptability does not predict job satisfaction at the interindividual level. We also discuss IV estimation in terms of its limits, weight the interpretation of its estimates against the temporality criterion for causal inference, and consider its possible extension to analyses of change.

## Introduction

People with high career adaptability report a wealth of desirable adaptation results, ranging from high career identity and calling to low turnover intentions, high employability, and high life satisfaction (for a meta-analysis, Rudolph et al., 2017). Career construction theory asserts that career adaptability—as a set of psychosocial resources that help people to manage their careers and ensure their social integration—explains why and how people with dispositions to adapt are more satisfied in several life domains (Savickas, 2002, 2005; Savickas et al., 2009). However, all life domains do not seem to benefit from career adaptability to the same extent. Specifically, career adaptability similarly predicts people’s satisfaction with their overall life and career, but recent meta-analytic results unexpectedly revealed no effect on job satisfaction when personality is controlled for (Rudolph et al., 2017). Therefore, it is important to place the relationship between career adaptability and job satisfaction under close scrutiny.

Our first objective in this article is to advance the understanding of this complex relationship. Specifically, we propose the existence of a simultaneous reverse effect of job satisfaction on career adaptability. By drawing on models and findings highlighting the affective ties of job satisfaction and career adaptability, we argue that affective dispositions of extraverted and neurotic individuals shape their job satisfaction, which in turn benefits their career adaptability. Because our argument implies that the results of regression techniques assuming unrelated predictors and error terms, such as ordinary least squares (OLS) regressions, might be biased, we also have a second methodological objective. Specifically, we aim to contribute to the broader accessibility and application of instrumental variable (IV) estimations in psychology. IV estimation, particularly two-stage least squares (2SLS) regression, is one possible solution to endogeneity concerns. Endogeneity characterizes “any case in which an explanatory variable may be correlated with the error term” (Wooldridge, 2009, p. 88). It can exist for a variety of reasons, including the omission of one or more variables or when simultaneous reverse causality exists (for a review, see Antonakis et al., 2014, Table 6.1). When endogeneity happens as in our case, the results of more traditional regression techniques are inconsistent, meaning that they do not capture the magnitude of the true population parameter. As a result, causal inference is hindered.

To achieve these two admittedly quite different objectives, we structure this article to offer readers the opportunity to easily focus on their interest. In the first part, we offer a reminder on how causal inference is ideally reached in quantitative research and then use algebra parsimoniously to show how endogeneity biases traditional OLS regression estimates and how 2SLS regression returns consistent estimates of an endogenous predictor on its outcome, provided that IVs—exogenous and relevant sources of variance—are available. In a second part, we empirically illustrate the potential of the IV method to remedy endogeneity threats. We present theoretical arguments and existing empirical evidence that link career adaptability and job satisfaction in one direction or the other. We outline how personality traits as possible IVs might help remedy the issue and compare the results obtained with the OLS and 2SLS regressions. Our results emphasize how confusing conclusions might be drawn if endogeneity issues are not accounted for. Finally, we discuss the theoretical implications of our results for research on career resources, as well as the opportunities and challenges of IV, particularly with respect to temporality in causal inference.

Overall, our examination of a simultaneous reverse effect of job satisfaction on career adaptability has the potential to make several contributions. From a methodological point of view, giving weight to a concrete empirical question makes the IV method more accessible to a non-specialized audience by allowing readers to focus on the illustration rather than the algebra. Using real data and constructs that are well-known in psychology and beyond (i.e., personality traits), our applied example complements the detailed descriptions of the IV method available elsewhere (Bascle, 2008; Podsakoff et al., 2012; Antonakis et al., 2014; MacKinnon and Pirlott, 2015). This is important given that the stakes at hand with the IV method have long been recognized and underlined (James, 1980; Bullock et al., 2010), and the models this method allows to test, corresponding to full mediations, are routinely depicted in psychology handbooks (Judd and Kenny, 2010, Figure 4.3). However, the use of the IV method is still scarce in psychology beyond a few transdisciplinary areas (for examples, Antonakis et al., 2014; Growiec and Growiec, 2014). From a theoretical point of view, our work provides opportunities to further link career construction theory with the literature on individuals’ affective resources and experiences. When testing career construction theory, scholars might under- or overestimate the effect of career adaptability if it is inconsistently estimated, thereby hindering theoretical advancements. Moreover, being able to recover consistent estimates might further help develop effective evidence-based interventions. This is an important step toward a better understanding of the scope of career construction theory and the development of meaningful practice.

## Inference of Causality

Causality has always been sought after in almost all scientific fields (Beebee et al., 2012; Hong, 2015; Pearl et al., 2016). However, causality may be the most difficult object to establish in scientific research. The reasons for such difficulty are directly related to the very notion of causality and to the confusion occurring between causality and other related but different concepts. The essential distinction to be made is between *association* and *causality*. Association, as quantified by correlation or similar tools, means that two observed phenomena are related. For instance, two psychological measures can tend to take simultaneously high or low values, or conversely, one measure can tend to take high values when the other takes low values. For instance, Brader (2005) conducted an experiment that demonstrated that the degree of enthusiasm shown in political ads is associated with the motivation viewers have to be involved in an election process. Even if there exists a strong link between two measures, association does not imply that the values taken by one measure are the reason for, or the cause of, the values taken by the other. On the other hand, the concept of causality expressly states that the values taken by one measure are (part of) the reason for observing specific values in the other.

Considering health and the need to put into evidence the determinants of sickness, Hill (1965) listed no less than nine elements that should be examined before being able to conclude that a relationship is causal. Currently, there is a consensus around three absolutely necessary conditions for a relationship to be causal (Kenny, 1979; Shadish et al., 2002): association, temporality, and the absence of a concurrent explanation. More precisely, to establish a causal relationship between phenomena A (the putative cause) and B (the observed effect), (1) A and B must be reliably associated, (2) A must precede B, and (3) there must be no other phenomenon that could also valuably explain the variations observed in B. It follows that association is not a synonym of causality but is a necessary condition for causality to exist.

While the first two conditions above can realistically be established, association being measured by correlation or similar indices and temporality being controlled for by a correct research design, this is not the case for the notion of concurrent explanations, which is the most difficult criterion to satisfy to make causal claims (Antonakis et al., 2014). First, these potential explanations must be identified; next, they must be measured, that is, they must be represented by a variable or a set of variables that we can use for analyses; and finally, a procedure that is able to demonstrate that they are not the real cause of effect B must be applied.

In psychology, this latter point is generally conducted via experiments (Shadish et al., 2002). The principle of a randomized experiment is to manipulate a condition or variable while controlling for other conditions through random assignment to demonstrate the effect of this particular condition on a response variable. This general setup can be used to show both that putative cause A indeed has an impact on effect B and that concurring phenomena do not have an impact on B. In the case of Brader’s (2005) study, the degree of enthusiasm shown in political ads was manipulated to study its relationship with the motivation of viewers to be involved in the election process. Combining a set of well-defined experiments with a strong association between A and B and the respect of temporality (A preceding B), the necessary conditions for demonstrating causality can then be met. However, having demonstrated causality is not necessarily the final goal of scientific research. It is also sometimes a necessary first step before moving to more specific relationships such as moderation and mediation (Shadish et al., 2002; Frazier et al., 2004).

Although the randomized experiment is viewed as a gold standard to demonstrate causality in psychology, other points of view still coexist, such as *robust dependence* (the association between cause A and effect B cannot be eliminated when controlling for possible confounders) and *generative process* (Goldthorpe, 2001). The latter, introduced first by Cox (1992), is especially interesting in that it opens a door for the use of more complex, and perhaps more realistic, modeling of causality. In substance, the idea is that a non-observable generating process is accountable for the causal relationship between A and B. Hence, manipulating only observable variables is not always sufficient for establishing causality (Blossfeld, 2009). Moreover, obtaining the necessary variables for an experiment is not always possible. When working with observational survey data, for instance, manipulating a variable is rarely feasible because the goal of such surveys is to obtain a representative picture of the population under study, so any influence on it must be seen as harmful. Other approaches must then be considered, and for regression models, the IV method discussed in this article is one possibility, allowing us to obtain consistent estimates of the relationship among phenomena by controlling for unobserved confounders (Stock and Watson, 2003; Wooldridge, 2009). In the case of more complex situations, for instance, with several dependent variables, other tools can also be mobilized, such as the structural causal model (Pearl, 2009).

## Endogeneity, Instrumental Variable Regression and Its Diagnostics

A major problem that can arise when conducting a regression analysis is the inconsistency of the estimates of the parameters of interest, that is, the non-convergence of these estimates toward their true but unknown value as the sample size moves to infinity. This issue may be caused by endogenous explanatory variables or, in other words, by predictors that are correlated with the error term (the part of the dependent variable that is not explained by predictors) of the regression. Endogeneity in the relationship between a presumed cause and its effect can stem from a large variety of issues, including omitted selection or variables (e.g., confounders and fixed effects), simultaneity (i.e., when explanatory and explained variables influence each other at the same time), measurement error, or common-source or common-method variance (for a review, see Antonakis et al., 2014, Table 6.1). In this case, the classical OLS procedure obtains inconsistent results (Stock and Watson, 2003). IV estimation is an interesting substitute due to its consistency in such cases. The procedure consists of identifying theoretically derived IVs, variables that can be considered exogenous to the model, to retrieve a consistent estimate for the relationship of interest for which the endogenous covariance is controlled for.

We now provide a brief explanation of why and how IV estimation—and, more specifically, the two-stage least square (2SLS) procedure—solves the problem of endogenous explanatory variables and present tests to compare the OLS and 2SLS regression estimates and examine whether theoretically derived exogenous variables are valid IVs from a statistical point of view. Although routinely applied in some social sciences such as economics and sociology (for reviews, see Angrist and Krueger, 2001; Bollen, 2012), the use of the IV method is still scarce in psychology beyond a few transdisciplinary areas at a crossroads with management (Antonakis et al., 2014; Bollmann and Krings, 2016) and positive psychology (Growiec and Growiec, 2014) for example. Readers interested in intuitive descriptions of endogeneity can refer to introductory articles and chapters (Bascle, 2008; Antonakis et al., 2014), and those interested in more detailed introductions can refer to handbooks in econometrics (e.g., Stock and Watson, 2003; Wooldridge, 2009) or to method books in sociology (e.g., Morgan and Winship, 2007).

### Inconsistency of OLS Regression

Consider a simple regression model with explained dependent variable *y* and a single independent regressor *x* (for observation *i* = 1, ……, *n*):

$${\text{y}}_{\text{i}}=\beta_{0}+\beta_{1}{\text{x}}_{\text{i}}+{\text{u}}_{\text{i}},$$

where β_0_ and β_1_are the intercept and the slope, respectively, and *u* is the error term following a *N(0,1)* distribution. The OLS estimator of β = [β_0_,β_1_] (Cameron and Trivedi, 2005, ch. 4.8) is as follows:

$$\hat{\beta_{\text{ols}}}={\left(\sum_{\text{i}=1}^{\text{n}}x_{\text{i}}x_{\text{i}}^{\text{T}}\right)}^{-1}\sum_{\text{i}=1}^{\text{n}}x_{\text{i}}y_{\text{i}}.$$

If there is a relationship (non-zero) between the error term and the independent variable or, in other words, when x is endogenous, $\hat{\beta_{\text{ols}}}$ becomes inconsistent (Cameron and Trivedi, 2005, ch. 4.8). The endogeneity can be mathematically described as follows:

corr(xu)≠0,

which means that the assumption of the independence between the regressor and the error term is violated. More specifically, $\hat{\beta_{\text{ols}}}$ includes both the indirect effect of *u* on *y* (through *x*) and the direct effect of *x* on *y*, while the aim of the OLS procedure is to measure only the direct impact of the independent variable *x* on *y*. One of the standard solutions to handle the problem of endogeneity is IV estimation, such as the 2SLS estimator, $\hat{\beta_{2\text{sls}}}$, instead of the common OLS procedure.

To check if there is an endogeneity problem, we recommend using the most powerful available procedure: the Hausman specification test (Hausman, 1978). It tests the estimates provided by both estimators ($\hat{\beta_{\text{ols}}}$ and $\hat{\beta_{2\text{sls}}}$), and it is implemented in all major statistical software programs (Croissant and Millo, 2008; Baum et al., 2010).

### Definition and Conditions of an Instrumental Variable

An instrument is an exogenous variable that takes its origin outside of the model and is unexplained by the model. The chosen IV for *x*, namely, *z*, is based on several conditions. The first condition, known as *instrument relevance*, is that there should be a significant degree of association between *z* and *x* for having a powerful instrument (Stock and Watson, 2003):

corr(zx)≠0.

This assumption can be verified by the underidentification test, which is based on the Lagrange Multiplier and the statistic provided by Kleibergen and Paap (2006). More specifically, consider the reduced-form regression for the case when only one variable is endogenous, namely, *x*_i_:

(1) $$x_{\text{i}}=\pi_{0}+\pi_{1}z_{\text{i}}+\xi_{\text{i}},$$

where *z* is the exogenous instrument, π_1_ is its coefficient, π_0_ is the slope and *ξ* is the error term following a *N(0,1)* distribution. In this case, the underidentification procedure tests the hypothesis that there is no significant association between *x* and *z*. If this hypothesis is rejected, then this first condition is satisfied. In other words, the test checks if π_1_ is significantly different from 0. If there are more than one endogenous variable, it is recommended that the test of the rank of the coefficient matrix be used (Cragg and Donald, 1993).

The second assumption, known as *instrument exogeneity*, states that *z* cannot be an explanatory variable of the model for *y* because, in such case, *z* would be correlated with the error term (Stock and Watson, 2003); thus,

corr(zu)=0.

When both conditions are satisfied, *z* can capture the exogenous changes in *x*. The general IV estimator is computed as follows:

$$\hat{\beta_{\text{IV}}}={\left(\sum_{\text{i}=1}^{\text{n}}z_{\text{i}}x_{\text{i}}^{\text{T}}\right)}^{-1}\sum_{\text{i}=1}^{\text{n}}z_{\text{i}}y_{\text{i}}.$$

In the case of a single regression model, the previous conditions are sufficient to define a valid instrument. However, the general case leads to the additional problems of identification. Consider the following multiple regression model with *K* independent variables, *M* of them being exogenous:

$$y_{\text{i}}=\beta_{0}+\beta_{1}x_{1\text{i}}+\beta_{2}x_{2\text{i}}+\cdots +\beta_{\text{K}}x_{\text{Ki}}+\psi_{\text{i}},$$

where ψ is the error term following an *N(0,1)* distribution. The order conditions, which are applied to have a consistent instrument, state that the number of instruments, *r*, must be at least equal to the number of endogenous variables, which means that *r ≥ K − M*.

The Sargan-Hansen test (Sargan, 1958; Hansen, 1982) is the most powerful method for checking overidentification (Hayashi, 2000). The hypothesis of this test is that the IVs are correlated with the residuals. If this is the case, the fit of the model is not good, and another combination of instruments must be found to obtain consistent estimates.

### 2SLS Estimator

The 2SLS estimator is one of the possible IV estimators that can be used. In econometrics, it is a commonly used estimator in the presence of endogeneity. This is due to the simplicity of the computation process and the consistency of this estimator. It consists of two stages. Suppose that *z* is a valid exogenous instrument for *x*. In the first stage, we regress *x* on *z* by using equation (1) and the standard OLS procedure. The predicted value of *x*_i_, $\hat{x_{\text{i}}}=\hat{\pi_{0}}+\hat{\pi_{1}}z_{\text{i}}$, is then used in the second stage, which corresponds to the standard OLS regression procedure of *y* on $\hat{x}$:

(2) $$y_{\text{i}}=\beta_{0}^{*}+\beta_{1}^{*}\hat{x_{\text{i}}}+\varepsilon_{\text{i}},$$

where ${\text{β}}_{0}^{*}$ is the intercept, ${\text{β}}_{1}^{*}$ is the slope, and ε is the error term following an *N(0,1)* distribution. The regression estimator of equation (2) is the so-called 2SLS estimator:

$$\hat{\beta_{2\text{sls}}}={\left(\sum_{\text{i}=1}^{\text{n}}\hat{x_{\text{i}}}{\hat{x_{\text{i}}}}^{\text{T}}\right)}^{-1}\sum_{\text{i}=1}^{\text{n}}\hat{x_{\text{i}}}y_{\text{i}},$$

where $\hat{x}$ is the predicted regressor from the first stage. If the provided instrument was correctly chosen, the resulting estimator of β is consistent. This procedure can be easily extended to the multiple regression case.

## Illustration

We now turn to a theoretically grounded illustration of the application of 2SLS regression in examining the relationship between career adaptability and job satisfaction. After briefly presenting career construction theory, its associated empirical evidence regarding the prediction of job satisfaction by career adaptability and the role of personality traits as potential IVs, we highlight an alternative complementary rationale, rooted in affect, suggesting that interindividual differences in job satisfaction might also partly explain career adaptability.

### How Career Adaptability Can Affect Job Satisfaction

Career adaptability refers to “individuals’ resources for coping with current and anticipated tasks, transitions and traumas in their occupational roles that, to some degree large or small, alter their social integration” (Savickas and Porfeli, 2012, p. 662). This self-regulatory, partly flexible competency encompasses four dimensions including concern, the planning and preparation of occupational steps; control, being decisive and taking responsibility for the evolution of one’s career; curiosity, the exploration of job-, career-, and self-related opportunities; and confidence, the conviction about one’s success in coping with occupational issues (Savickas, 2005). Career construction theory posits that people differ in their readiness (e.g., personality traits) and ability (e.g., career adaptability) to behave in ways that facilitate their adaptation, namely, their social integration into and satisfaction with various life domains (Savickas, 2002, 2005; Savickas et al., 2009). Career adaptability thus represents an important mechanism in this theory, explaining why and how, over an extended period of time, people with specific dispositions to adapt are more satisfied with various life domains.

Compared to others, individuals with higher career adaptability report a wealth of positive job- and career-related experiences (for a review, see Johnston, 2018; for meta-analytical evidence, see Rudolph et al., 2017). For example, they experience less job content plateaus (Jiang, 2016), have less intention to change jobs (Chan and Mai, 2015) and have more entrepreneurial intentions than people with lower career adaptability (McKenna et al., 2016). In a similar vein, unemployed people who feel more in control of and confident about their career-related decision making also find higher-quality jobs (Koen et al., 2010).

Despite these associations with multiple desirable job- and career-related indicators, career adaptability does not seem to benefit satisfaction with all life domains to the same extent and, in particular, with job satisfaction. Individuals with higher career adaptability are more satisfied with their life and report higher levels of general well-being than those with lower career adaptability (e.g., Hirschi, 2009; Maggiori et al., 2013; Ginevra et al., 2018). They are also more satisfied with their careers than others (e.g., Zacher, 2014; Haibo et al., 2018). However, the findings depict a different and conflicting picture regarding job satisfaction. While several studies also found that it could be predicted by career adaptability (e.g., Maggiori et al., 2013; Han and Rojewski, 2014; Zacher and Griffin, 2015), meta-analytical results suggest a weaker effect than for life and career satisfaction (Rudolph et al., 2017). Moreover, when personality traits were controlled for, career adaptability predicted people’s satisfaction with their overall life and career but not their satisfaction with their jobs. This led Rudolph et al. (2017) to call for further research on this surprising finding.

Assuming the existence of a simultaneous reverse relationship (as described in the next section), one would argue that career adaptability is endogenous in predicting job satisfaction. As a result, statistical analyses that assume that career adaptability is not related to the error term of the equation predicting job satisfaction (e.g., OLS regression, maximum likelihood estimation) would yield inconsistent estimates. In such cases and more generally when experiments cannot be considered, the IV method constitutes one possible solution to recover a consistent estimate.

**FIGURE 1:**
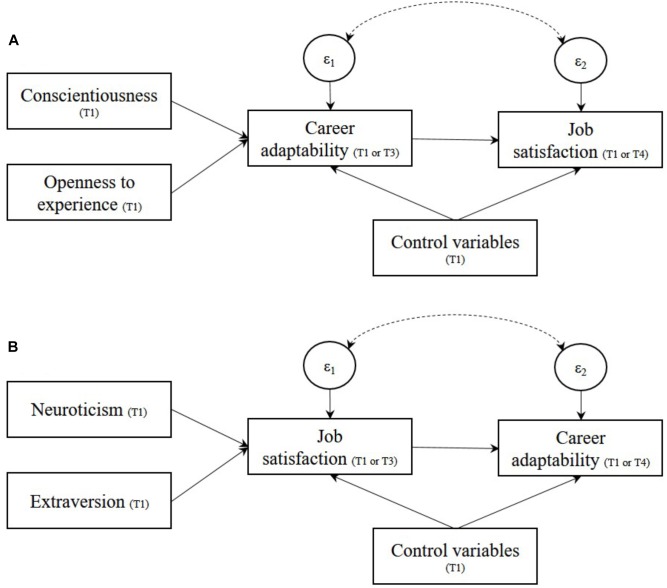
Alternative models tested in the empirical illustration with 2SLS regressions. **(A)** Depicts how career adaptability can affect job satisfaction. **(B)** Depicts how job satisfaction might affect career adaptability. For the sake of clarity, correlations between exogenous variables are omitted and control variables are grouped. ε_1_ and ε_2_ represent error terms of the 1st and 2nd stage equations, respectively. Endogeneity corresponds to the correlation represented by the dashed line between the error terms; this correlation is controlled for by the 2SLS regression.

Since personality traits are a part of people’s readiness to adapt and partly inheritable and genetically determined (Jang et al., 1998), they can theoretically and empirically be considered relevant and exogenous sources of variance. As maintained by career construction theory and evidence, career adaptability relates to several personality traits (Rudolph et al., 2017). Individuals scoring high in conscientiousness, openness to experience, and extraversion and low in neuroticism present high career adaptability scores (Rossier et al., 2012; Rudolph et al., 2017). In particular, conscientiousness and openness to experience are consistently related to career resources (e.g., Teixeira et al., 2012; van Vianen et al., 2012; Zacher, 2016), and career adaptability also partially explains why highly conscientious people are engaged at work (Rossier et al., 2012). In contrast, the relationships between career adaptability and neuroticism and extraversion are inconsistent. For example, when considered simultaneously with other personality traits, neuroticism and extraversion do not always predict career adaptability (e.g., Li et al., 2015; Zacher, 2016). In sum, as depicted in Figure 1A, career construction theory and the related empirical findings suggest that conscientiousness and openness to experience are positively related to career adaptability, which in turn predicts job satisfaction.

### How Job Satisfaction Might Affect Career Adaptability

In contrast, or perhaps complementarily, models highlighting the affective origins of job satisfaction and its influence on broader life domains, together with those tying career adaptability to affective experiences, suggest that job satisfaction might also predict career adaptability. As “a positive (or negative) evaluative judgment one makes about one’s job or job situation” (Weiss, 2002, p. 175), job satisfaction has long been viewed not only as a cognitive but also as an affectively laden attitude. The integrative model of satisfaction with life domains, for example, emphasizes the role of the affective tendencies underlying neuroticism and extraversion in explaining job satisfaction and outcomes in one’s broader life domains (Heller et al., 2004). According to this model, neurotic and extraverted people consistently expose themselves and experience congruent affective circumstances in their life domains, such as at their workplace. In turn, emotional states shape the general judgment of these domains and impact life satisfaction (Weiss and Cropanzano, 1996). Meta-analytical findings show that affective predispositions, such as neuroticism and extraversion, as well as negative and positive affective traits and states, are indeed related to people’s satisfaction with their jobs (Connolly and Viswesvaran, 2000; Judge et al., 2002; Thoresen et al., 2003).

Career adaptability has also been theoretically related to affective experiences. In Rossier’s (2015) model, career adaptability influences individuals’ adapting responses and can be activated by experiences originating from the combination of individual predispositions and the broader context (Rossier, 2015; Rossier et al., 2017). In this model, even though career adaptability may constitute a protective factor against negative experiences, when people are confronted with long-term negative states and experiences, their job attitudes suffer from them on the long term (Fiori et al., 2015). Meta-analytic evidence confirmed that threatening working conditions are strongly connected to negative job attitudes via affect (Podsakoff et al., 2007; Zhao et al., 2007). Moreover, Johnston (2018) noted in her review that some affectively laden events or experiences appraised as threatening rather than challenging might drain career adaptability. For instance, people who experience job insecurity and strain concurrently report lower career adaptability than those who do not experience these situations (Maggiori et al., 2013). As such, people who experience more negative job attitudes, such as those who are neurotic or less extraverted, might well report lower career adaptability. Taken together, these findings suggest that neuroticism and extraversion represent negative and positive predictors of job satisfaction, respectively, which in turn positively affect career adaptability (Figure 1B).

These two lines of theoretical arguments and their associated empirical evidence simultaneously suggest that career adaptability (originating in conscientiousness and openness to experience) is an antecedent of job satisfaction and/or that it is the other way around, that is, job attitudes stemming from affective predispositions influence individuals’ level of career adaptability. Importantly, if both effects exist, each of them would suggest the presence of endogeneity in the prediction of the second stage of the other model (as depicted by the correlated error terms in the figures). Therefore, these not mutually exclusive models remain to be thoroughly tested and constitute fertile ground to illustrate how 2SLS regressions can solve potential endogeneity issues.

## Materials and Methods

We tested these models, capitalizing on the specific design of the Professional Paths Survey and the differential relationships that personality maintains with career adaptability and job satisfaction. Namely, in this study, personality traits were assessed in 2012 (T1), and career adaptability and job satisfaction were measured in 2012 (T1), 2014 (T3), and 2015 (T4; career adaptability was not measured in 2013). This offered the opportunity to investigate a cross-sectional (with T1 predictors and outcomes) and time-lagged effect (with T3 predictors and T4 outcomes) based on the same sample but different measurement points. With respect to causal claims regarding the relationship between career adaptability and job satisfaction, this study would allow us to satisfy the criterion of association (i.e., observing a significant relationship) and the criterion of temporality (i.e., by design for the time-lagged models) and statistically control for concurring causes, thereby ruling out numerous threats to causal inference. However, as discussed below, other factors may still have to be considered before making strong causal claims.

### Procedure

The data come from three waves of a panel study on the professional trajectories of the Swiss working-age population. Participants yearly received an invitation letter reminding them of the goals and relevance of the project and providing them with instructions on how to participate. They could complete the survey in German or French and in various formats (i.e., depending on the survey parts, a computer-assisted telephone interview, an online self-administered questionnaire, or a paper-pencil questionnaire). Sixty-four percent of the sample participated in German, and depending on the wave, between 82.6 and 88.9% of the sample completed the survey with the online questionnaire. Data were matched with a 6-digit identifier, accompanied by a 4-digit password given in the letter.

### Participants

The sample stemmed from a wider random sample drawn from national registers in Switzerland. From the 7,030 valid addresses contacted, 3,049 participants completed, at least partially, one wave (response rate of 43.37%). For this study, we kept a subsample of 836 participants without missing data on any of the study variables (see below). This subsample was slightly older than the full sample [*M*_age(study)_ = 43.04 years, *M*_age(full sample)_ = 41.34, *t*(2,467) = 4.64, *p* < 0.001, *d* = 0.20], but it did not differ from the full sample with respect to gender [females _study_ = 51.3%, females _full sample_ = 51.7, χ^2^(1) = 0.02, *p* = 0.90]. More detailed information on the sampling procedure and data collection is available elsewhere (Maggiori et al., 2016).

### Measures

#### Personality

The five personality traits of neuroticism, extraversion, openness to experience, agreeableness, and conscientiousness were assessed with the validated German and French versions of the NEO Five-Factor Inventory Revised (Schmitz et al., 2001; Aluja et al., 2005). At T1, participants described themselves, indicating their degree of agreement with the 60 items on a 5-point scale, ranging from 1 = *strongly disagree* to 5 = *strongly agree*.

#### Career Adaptability

The Career Adapt-Abilities Scale-Short Form (CAAS-SF) was used to evaluate participants’ career adaptability (Maggiori et al., 2017). At T1, T3, and T4, participants rated how much they disposed of resources to manage their professional path on a 5-point scale (1 = *I do not have the ability to…/This is not a resource for me*, 5 = *I have a very strong ability to…/This is a very important resource for me*). The twelve items of the CAAS-SF are equally distributed into resources of concern (e.g., Thinking about what my future will be like), control (e.g., Making decisions by myself), curiosity (e.g., Looking for opportunities to grow as a person), and confidence (e.g., Taking care to do things well).

#### Job Satisfaction

Job satisfaction was measured with six items at T1, T3, and T4. One item consisted of a global assessment (i.e., “Overall, to what extent are you satisfied with your job?”), and the remaining five items were adapted from the brief version of the *Minnesota Satisfaction Questionnaire* (Weiss et al., 1967) to assess participants’ satisfaction in their current job with their supervisor, job security, salary, working conditions, and colleagues. The response scale ranged from 1 = *not at all satisfied* to 4 = c*ompletely satisfied.*

### Data Analysis Strategy

We first demonstrated through a series of confirmatory factor analyses that job satisfaction and career adaptability are distinct. Given that job satisfaction and career adaptability were measured at three time points, we also assessed their longitudinal measurement invariance to ensure their comparability over time. Additionally, separate exploratory structural equation models were computed for personality traits (Marsh et al., 2010). In proceeding with our illustration, we concentrated, in the first set of analyses, on the effect of career adaptability on job satisfaction, which we tested with two 2SLS regression models, one with a cross-sectional path from T1 career adaptability to T1 job satisfaction and one with a time-lagged path from T3 career adaptability to T4 job satisfaction. Conscientiousness and openness to experience were selected here as IVs based on the respective relationship these traits maintain with career adaptability (see Figure 1A). That is, they were used to predict the values of career adaptability in the first stage of these 2SLS regressions. Then, in the second stage, job satisfaction was regressed on the predicted values of career adaptability, thus yielding a consistent estimate of the influence of career adaptability on job satisfaction. In these models, the remaining personality traits—neuroticism, extraversion, and agreeableness—were entered as control variables, along with participants’ gender and age. For all models, we report the results obtained with OLS regressions to highlight their similarities and differences with the results of 2SLS regressions. We also describe the diagnostic tests in simple terms and present their results and interpretation.

We followed a similar procedure to examine the reverse relationship, namely, the cross-sectional and time-lagged effect of job satisfaction on career adaptability, in a second set of analyses. Here, based on the arguments that people’s career adaptability could be shaped by their job satisfaction because of the affective tendencies reflected in neuroticism and extraversion, we selected these two personality traits as IVs (see Figure 1B). That is, in these 2SLS regressions, neuroticism and extraversion predicted job satisfaction in the first stage. The predicted values of job satisfaction were then used as predictors of career adaptability in the second stage. Again, the remaining personality traits—conscientiousness, openness to experience, and agreeableness—were entered as control variables, along with participants’ gender and age.

This second set of models is not mandatory for the first set to be complete, and vice versa. As mentioned above, reverse causality is only one possible source of endogeneity (Antonakis et al., 2014); if the suspected source of endogeneity was, for instance, an omitted confounding variable (e.g., intelligence), testing a reverse model would not be warranted. We conducted this second set of analyses because, from a substantive point of view, our theoretical arguments might complement or compete with the argument of the first model. This second set of analyses additionally points at the reverse relationship as the likely source of endogeneity that is responsible for the contradictory results obtained between the OLS and 2SLS regressions in the first set of analyses. Finally, it illustratively repeats the steps and diagnostics of the IV approach and highlights the contrasting results obtained for each hypothesized direction of the relationship.

Several statistical packages propose functions to estimate 2SLS regressions. In Stata 12—the package used here—2SLS regressions are available through the *ivreg2* command (Baum et al., 2010). This command automatically provides a series of statistics (and their *p*-values), which are diagnostics of the quality of the 2SLS regression model. The Hausman test is obtained with the *ivendog* command, following the *ivreg2* command. The *ivreg2* package can be downloaded through the Stata interface. As an illustration, we provide Stata 12, Mplus 7, SPSS, and R syntaxes and information regarding the necessary packages to test the model presented in Figure 1A in the Supplementary Material.

## Results

Table 1 lists the means, standard deviations, correlations, and reliabilities of the scales.

**Table 1:** Means, standard deviations, correlations, and reliabilities of study variables.

Variable	*M*	*SD*	1	2	3	4	5	6	7	8	9	10	11	12	13
(1) Gender^a^	51.3%	–	–												
(2) Birth year (in years)	1968.95	8.22	−0.01	–											
(3) Neuroticism	2.60	0.53	**0.18**	**0.12**	(0.83)										
(4) Extraversion	3.40	0.48	**0.10**	0.02	**−0.32**	(0.76)									
(5) Conscientiousness	3.93	0.43	0.06	−0.04	**−0.29**	**0.27**	(0.77)								
(6) Openness to experience	3.47	0.50	**0.16**	**−0.10**	−0.01	**0.31**	0.07	(0.76)							
(7) Agreeableness	3.42	0.30	**0.13**	−0.08	−0.01	**0.12**	0.03	**0.19**	(0.70)						
(8) T1 Career adaptability	3.76	0.50	−0.00	−0.05	**−0.38**	**0.35**	**0.45**	**0.38**	0.03	(0.88)					
(9) T3 Career adaptability	3.76	0.51	−0.00	−0.01	**−0.30**	**0.29**	**0.36**	**0.33**	0.01	**0.64**	(0.89)				
(10) T4 Career adaptability	3.79	0.55	0.02	−0.04	**−0.28**	**0.27**	**0.36**	**0.33**	0.01	**0.59**	**0.66**	(0.91)			
(11) T1 Job satisfaction	3.23	0.43	0.02	−0.04	**−0.29**	**0.23**	**0.15**	0.07	**0.10**	**0.21**	**0.13**	**0.11**	(0.073)		
(12) T3 Job satisfaction	3.18	0.46	0.01	−0.03	**−0.26**	**0.11**	0.06	0.03	0.09	**0.20**	**0.21**	**0.17**	**0.49**	(0.75)	
(13) T4 Job satisfaction	3.16	0.47	0.01	−0.05	**−0.25**	**0.15**	0.06	0.06	**0.13**	**0.17**	**0.18**	**0.25**	**0.43**	**0.59**	(0.76)

*N = 836. Correlations significant at p < 0.01 are printed in bold. Reliability coefficients (alphas) are shown in parentheses along the diagonal. ^a^For gender, the percentage rather than the mean is displayed.*

### Confirmatory Factor Analysis and Longitudinal Measurement Invariance

Our confirmatory factor analyses compared, for each time point, a measurement model, with all indicators loading on a single latent variable, to another one, with indicators loading on two correlated latent variables, representing job satisfaction and career adaptability. We used a robust maximum likelihood estimator to consider a possibly skewed distribution of the data. Three indicator parcels were formed for job satisfaction by progressively averaging items with the strongest and weakest factor loadings (i.e., the item-to-construct balance principle; Little et al., 2002). Given the existing evidence for the second-order latent structure of career adaptability (Maggiori et al., 2017) and our interest in its overall scores, we formed four indicators of career adaptability by averaging the three items measuring each of its dimensions. Parcels present advantageous psychometric (e.g., higher ratio of common-to-unique factor variance and fewer chances of distributional violations) and estimation characteristics (e.g., parsimony and lower likelihood of correlated residuals between parcels than between items) when the primary focus of a study lies on relations between variables (Little et al., 2002). We evaluated and compared the models, relying on cutoff values >0.95 for the comparative fit index (CFI), <0.06 for the root mean square error of approximation (RMSEA), and <0.08 for the standardized root mean square residual (SRMR) to indicate model fit (Hu and Bentler, 1999), as well as on the Satorra–Bentler scaled χ^2^ difference test because robust estimation biases original χ^2^ values (Satorra and Bentler, 2001). For all three time points, the two-variable measurement models revealed excellent fit indices, which were superior to the one-variable models (Table 2). This implied that the job satisfaction and career adaptability measures effectively captured distinct constructs at all three time points. The results regarding the personality traits were very consistent with, yet weaker than, those found by Marsh et al. (2010); see Supplementary Material for details.

**Table 2:** Confirmatory factor analyses of job satisfaction and career adaptability for the three time points.

Time, models	SB-χ^2^	SB-C	*df*	*p*-value	CFI	RMSEA [90% C.I.]	SRMR	SB-Δχ^2^ (*df*), *p*-value
*Time 1*								
One-variable model	423.842	1.1661	14	<0.001	0.673	0.187 [0.172;0.203]	0.125	
Two-variable model	25.636	1.1793	13	0.019	0.990	0.034 [0.013;0.053]	0.025	466.575 (1), *p* < 0.001
*Time 3*								
One-variable model	544.770	1.1281	14	<0.001	0.643	0.213 [0.198;0.228]	0.139	
Two-variable model	33.454	1.1077	13	0.002	0.986	0.043 [0.025;0.062]	0.025	414.482 (1), *p* < 0.001
*Time 4*								
One-variable model	566.516	1.1226	14	<0.001	0.694	0.217 [0.202;0.233]	0.140	
Two-variable model	12.659	1.0851	13	0.475	1.000	0.000 [0.000;0.034]	0.015	386.457 (1); *p* < 0.001

*N = 836. SB-χ^2^, With robust maximum estimation, χ^2^ cannot be used as it is for difference test, and must be corrected using the Satorra–Bentler correction factor; SB-C, Satorra–Bentler correction factor; df, degrees of freedom; CFI, comparative fit index; RMSEA, root mean square error of approximation; SRMR, standardized root mean square residual; SB-Δχ^2^, Satorra–Bentler scaled χ^2^ difference test.*

Second, to ascertain the comparability over time of our various cross-sectional and time-lagged models, we examined the longitudinal measurement invariance of job satisfaction and career adaptability (Vandenberg and Lance, 2000; Chen, 2007). When comparing progressively and more strictly invariant models, we referred to changes in fit indices using the cutoff values recommended by Chen (2007); when (*N* > 300, non-invariance would be indicated mainly by ΔCFI ≥-0.010, supplemented by ΔRMSEA ≥ 0.015 and by ΔSRMR ≥ 0.030 for loading invariance, and ΔSRMR ≥ 0.010 for intercept or residual invariance) because the χ^2^-difference test is commonly viewed as very sensitive to the sample size (Cheung and Rensvold, 2002; Chen, 2007; Little et al., 2007). Considering ΔCFI as the main criterion (Chen, 2007), the results (Table 3) revealed only small changes in the fit indices, which remained excellent across all levels of invariance, despite a ΔSRMR slightly above the threshold for strict (residual) invariance. This implied that at least the scalar invariance of job satisfaction and career adaptability could be assumed.

**Table 3:** Results of longitudinal measurement invariance for job satisfaction and career adaptability over a 3-year interval.

Invariance type	SB-χ^2^	SB-C	*df*	*p*-value	CFI	RMSEA [90% CI]	SRMR	ΔCFI	ΔRMSEA	ΔSRMR
Configural invariance	197.911	1.061	153	0.008	0.994	0.019 [0.010;0.026]	0.030	–	–	–
Metric invariance (loading)	202.258	1.062	163	0.020	0.995	0.017 [0.007;0.024]	0.032	0.001	0.002	0.002
Scalar invariance (intercept)	254.157	1.058	173	<0.001	0.989	0.024 [0.017;0.030]	0.034	0.006	0.007	0.002
Strict invariance (residual)	285.400	1.067	187	<0.001	0.987	0.025 [0.019;0.031]	0.048	0.002	0.001	0.012

*N = 836. df, degrees of freedom; SB-C, Satorra-Bentler correction factor; CFI, comparative fit index; RMSEA, root mean square error of approximation; SRMR, standardized root mean square residual.*

### Career Adaptability → Job Satisfaction

We start with the set of analyses to identify the cross-sectional explanation of T1 job satisfaction by T1 career adaptability. Here, the first stage of the 2SLS regression revealed that, even after controlling for participants’ gender, age, neuroticism, extraversion, and agreeableness, T1 career adaptability was significantly predicted by the IVs, namely, conscientiousness, *b* = 0.39, *p* < 0.001, and openness to experience, *b* = 0.35, *p* < 0.001. Together, these two personality traits explained a significant part of the variance in T1 career adaptability, *F*(2,828) = 140.49, *p* < 0.001, partial *R*^2^ = 0.25. That is, the more conscientious and open to experience the individuals were, the higher their career adaptability. In line with meta-analytic results (Rudolph et al., 2017) but contrary to previous studies, the second stage of this 2SLS regression model indicated that, when instrumented with conscientiousness and openness to experience, T1 career adaptability did not significantly predict T1 job satisfaction (Table 4, first column). In contrast to these results, those obtained with OLS regressions yielded an estimate for T1 career adaptability that was positive and similar in magnitude (i.e., small) but significant (Table 4, second column). Thus, contrary to the 2SLS regression results consistent with meta-analytic estimates, those obtained with OLS regressions indicated that individuals with higher career adaptability also had higher job satisfaction, pointing toward a conclusion similar to those of previous studies.

**Table 4:** Results of 2SLS and OLS regressions predicting job satisfaction on cross-sectional and longitudinal data.

	T1 Job satisfaction		T4 Job satisfaction	
	2nd stage		2nd stage
	2SLS	OLS	2SLS	OLS
*Control variables*				
Agreeableness	0.112^∗^	0.117^∗^	0.183^∗∗∗^	0.194^∗∗∗^
Neuroticism	−0.187^∗∗∗^	−0.177^∗∗∗^	−0.214^∗∗∗^	−0.191^∗∗∗^
Extraversion	0.103^∗∗^	0.104^∗∗^	0.061	0.046
Gender^a^	0.031	0.032	0.033	0.038
Birth year	−0.000	−0.000	−0.001	−0.001
*Instrumental variables*				
Conscientiousness		0.011		−0.083^∗^
Openeness to experience		−0.024		−0.030
*Endogenous predictors*				
T1 Career adaptability	0.054	0.076^∗^		
T3 Career adaptability			−0.035	0.130^∗∗∗^
Constant	3.144	3.272	4.085	4.523
*F*	17.49	13.74	12.4	11.35
*df*	6, 829	8, 827	6, 829	8, 827
(pseudo-)*R^2^*	0.12	0.12	0.07	0.10

*N = 836. ^∗^p < 0.05; ^∗∗^p < 0.01; and ^∗∗∗^p < 0.001. Unstandardized regression weights are presented. ^a^1 = male, 2 = female.*

The three statistic diagnostics of the 2SLS regression model and their related *p*-values guide the interpretations of the differences observed in the results compared to those of OLS regression. First, the underidentification test was significant, χ^2^(2) = 211.81, *p* < 0.001, suggesting that our instruments (i.e., conscientiousness and openness to experience) were significantly correlated with T1 career adaptability. Second, the Sargan-Hansen statistic and its *p*-value, χ^2^(1) = 0.65, *p* = 0.42, suggested that it was reasonable to exclude conscientiousness and openness to experience from the prediction of T1 job satisfaction in the second stage of the 2SLS regression. Based on these tests, the selection of conscientiousness and openness to experience as IVs was empirically grounded. Finally, the Hausman test for this model was not significant, χ^2^(1) = 0.09, *p* = 0.76. This indicated that endogeneity was not biasing the estimate of the effect of T1 job satisfaction on T1 career adaptability in a problematic way. In this situation, it is usually recommended to trust the results obtained with OLS regression since 2SLS regressions can sometimes yield less precise coefficients (we discuss this point further below; Baum, 2009).

Turning to the time-lagged effect of T3 career adaptability on T4 job satisfaction, conscientiousness, *b* = 0.33, *p* < 0.001, and openness to experience, *b* = 0.32, *p* < 0.001, had a positive and significant relationship with T3 career adaptability in the first stage of the 2SLS regression, *F*(2,828) = 85.22, *p* < 0.001, partial *R*^2^ = 0.17. Thus, similar to the results of the cross-sectional analysis, more conscientious and open individuals also saw themselves as more adaptable in their career two years later. For this model, the underidentification test was significant, χ^2^(1) = 142.71, *p* < 0.001, and the Sargan-Hansen test was non-significant, χ^2^(1) = 1.04, *p* = 0.31. This suggested that it was meaningful to use these IVs to predict T3 career adaptability and exclude them from the prediction of T4 job satisfaction. The consistent estimate for the effect of T3 satisfaction on T4 job satisfaction was, however, negative and non-significant when computed with the 2SLS regression, whereas its OLS regression counterpart was positive and significant (Table 4, columns 3 and 4). The Hausman test revealed a non-significant *p*-value, χ^2^(1) = 3.72, *p* = 0.054. However, this value was near the conventional levels of significance, suggesting potential bias in the 1-year lagged effect of career adaptability on job satisfaction.

In sum, firm conclusions were difficult to draw. Although endogeneity did not appear to be a threat to the cross-sectional model, the Hausman test conducted on the time-lagged model suggested the presence of possible bias, despite the conventional level of significance not being reached. We recommend, here, to not be too strict with the significance threshold because theoretical and empirical arguments both tend to speak for the presence of endogeneity. Thus, these results do not seem to offer evidence of an effect. At best, the magnitude of the overall 1-year lagged relationship between career adaptability and job satisfaction is small.

### Job Satisfaction → Career Adaptability

Turning to the set of analyses examining the cross-sectional effect of T1 job satisfaction on T1 career adaptability, a different picture emerged. Accounting for participants’ gender, age, conscientiousness, openness to experience, and agreeableness, T1 job satisfaction was significantly predicted by the IVs selected, namely, neuroticism, *b* = −0.19, *p* < 0.001, and extraversion, *b* = 0.11, *p* = 0.001, *F*(2,828) = 36.59, *p* < 0.001, partial *R*^2^ = 0.08. Thus, more neurotic individuals were concurrently less satisfied with their jobs, whereas more extraverted individuals were more satisfied with their jobs. The second stage of the 2SLS regression model revealed that, when instrumented, T1 job satisfaction was positively and significantly related to T1 career adaptability (Table 5, column 1). The diagnostics revealed that the assumptions underlying the use of neuroticism and extraversion as IVs were plausible, underidentification test χ^2^(2) = 67.88, *p* < 0.001, Sargan-Hansen test χ^2^(1) = 0.84, *p* = 0.36. According to the Hausman test, the results of OLS regressions would yield a biased estimate for T1 satisfaction, χ^2^(1) = 69.96, *p* < 0.001. As shown in Table 5, columns 1 and 2, a large difference in the magnitude of this estimate existed between the OLS and 2SLS regressions. Although the *R*^2^ values obtained with 2SLS regressions are not truly informative (Wooldridge, 2009), it is possible to compute a pseudo-*R*^2^ based on the correlation between the observed and predicted values of career adaptability purged from bias; this yielded a value of 17%. Job satisfaction can be seen as one factor that concurrently accounts for the career adaptability of less neurotic and more extraverted individuals.

**Table 5:** Results of 2SLS and OLS regressions predicting career adaptability on cross-sectional and longitudinal data.

	T1 Career adaptability		T4 Career adaptability	
	2nd stage		2nd stage
	2SLS	OLS	2SLS	OLS
*Control variables*				
Agreeableness	−0.210^∗∗^	−0.095^∗^	−0.213^∗∗^	−0.134^∗^
Conscientiousness	0.343^∗∗∗^	0.386^∗∗∗^	0.392^∗∗∗^	0.365^∗∗∗^
Openeness to experience	0.333^∗∗∗^	0.346^∗∗∗^	0.351^∗∗∗^	0.339^∗∗∗^
Gender^a^	−0.072	−0.035	−0.051	−0.020
Birth year	0.001	0.001	0.001	0.001
*Instrumental variables*				
Neuroticism		−0.222^∗∗∗^		−0.159^∗∗∗^
Extraversion		0.072^∗^		0.059
*Endogenous predictors*				
T1 Job satisfaction	1.082^∗∗∗^	0.070^∗^		
T3 Job satisfaction			0.866^∗∗∗^	0.129^∗∗∗^
Constant	−3.718	−0.934	−3.804	−1.358
*F*	44.02	70.9	34.55	39.61
*df*	6, 829	8, 827	6, 829	8, 827
(pseudo)-*R^2^*	0.17	0.41	0.15	0.28

*N = 836. ^∗^p < 0.05; ^∗∗^p < 0.01; and ^∗∗∗^p < 0.001. Unstandardized regression weights are presented. ^a^1 = male, 2 = female.*

Consistent with the cross-sectional results, the 1-year lagged effect of T3 job satisfaction on T4 career adaptability also revealed positive and significant results (Table 5, column 3). Computing a pseudo-*R*^2^, this model explained 15% of the variance in T4 career adaptability. Here, T3 job satisfaction was significantly and negatively predicted by T1 neuroticism, *b* = −0.24, *p* < 0.001, but not T1 extraversion, *b* = 0.02, *p* = 0.65, *F*(2,828) = 30.41, *p* < 0.001, partial *R*^2^ = 0.07. That is, more neurotic individuals are less satisfied with their jobs than less neurotic individuals 2 years later. Despite the non-significant relationship of extraversion, the 2SLS regression diagnostics suggested that it was reasonable to use these IVs together to predict T3 job satisfaction and exclude them from the prediction of T4 career adaptability, underidentification test, χ^2^(2) = 57.20, *p* < 0.001, Sargan-Hansen test, χ^2^(1) = 1.00, *p* = 0.317. In addition, the estimate for T3 job satisfaction obtained with OLS regression differed significantly from the more consistent estimate obtained with 2SLS regression, as suggested by the Hausman test χ^2^(1) = 25.82, *p* < 0.001 (compare Table 5, columns 3 and 4). Overall, the results of the 2SLS regressions conducted on the time-lagged effect of job satisfaction on career adaptability were very consistent with the cross-sectional results but differed from those obtained using the OLS regressions. These results speak in favor of job satisfaction as one factor that contributes to the career adaptability resources that people have at their disposition concurrently and one year later.

### Discussion of Results for Career Adaptability Research

Overall, the OLS regressions estimates indicated that, with personality remaining constant, career adaptability predicted job satisfaction concurrently and with a time lag, albeit weakly, which was consistent with the results of previous cross-sectional and longitudinal research (e.g., Fiori et al., 2015; Zacher and Griffin, 2015). However, when this relationship was instrumented with conscientiousness and openness to experience to account for potential endogeneity in career adaptability, career adaptability did not affect job satisfaction concurrently or over time. It is crucial to note that these later results were in line with the non-significant meta-analytic effect of career adaptability on job satisfaction, above and beyond personality traits (Rudolph et al., 2017). They highlighted the power of IV estimation in approaching population-wide estimates. Additional analyses revealed that, when instrumented with neuroticism and extraversion, the relationship of job satisfaction with career adaptability emerged as significant and stronger in magnitude than the reverse effect, both concurrently and over time. These results are consistent with the established relationships between personality-based affective dispositions and job satisfaction (e.g., Judge et al., 2002) and with recent findings that point to ties between individual differences in career adaptability and affective states (Fiori et al., 2015).

Interestingly, endogeneity appeared to bias only one direction of the career adaptability-job satisfaction relationship. As such, it unlikely stemmed from common method variance, one of the documented sources of endogeneity (Antonakis et al., 2014). If this were the case, analyses for both directional associations would have yielded a significant result of the endogeneity test. This finding might suggest that part of the variance that was purged from the 2SLS regressions might in fact be substantial. This highlights the importance of having clear expectations regarding the source of endogeneity and not merely accounting for it. Moreover, this finding points toward complex substantial dynamics between personality traits, job experiences and attitudes, as well as career adaptability (Rossier et al., 2017). Clearly, more research in this area is warranted to fully understand the complex interplay between career adaptability and affect. Affective states and attitudes at work experienced by neurotic and extroverted individuals might well play a role in the activation or accumulation of adaptability resources, originating in conscientiousness and openness to experience. Aversive affective events may, for example, activate or consume the career resources needed to show adaptive responses and outcomes (Savickas, 2005; Johnston, 2018). Different from our interindividual perspective, future efforts might therefore investigate how situational and environmental characteristics and personal resources interact in shaping intraindividual changes in adaptation (Rossier et al., 2017; Urbanaviciute et al., 2018).

## General Discussion

Instrumental variable estimation represents an alternative set of regression analyses that have the potential to provide consistent estimates, despite threats of endogeneity. We illustrated its application, with 2SLS regressions, building on the differential relationships that broad personality traits maintain with career adaptability and job satisfaction to examine the most plausible direction of the relationships among these constructs in the broader population. Overall, this research is informative in at least three points. First, as elaborated above, we showed that endogeneity can at times significantly bias OLS regression estimates and seriously hinder inference. Second, IV estimation has the potential to overcome this problem, provided that instruments of quality are selected. Under certain conditions, then, threats to causal inference can be reduced. Nevertheless, as a third point, when interpreting the IV estimation results, researchers should carefully consider the implications of their research design and analyses for causal claims; in particular, we consider here two issues related to time.

### Instrumental Variables: Conditions, Opportunities, and Limits

One promise—and perhaps also challenge—in using IV estimation lies in the identification of meaningful and theoretically derived IVs (Podsakoff et al., 2012). In psychology, IVs can be found among genetically based and relatively stable individual differences (e.g., personality traits), events outside the influence zone of participants, or natural experiments (for a fuller discussion, see Bollen, 2012). Importantly, however, to qualify as IVs, variables must satisfy two conditions: they must be relevant, namely, significantly correlated with the endogenous explanatory variables, and exogenous, that is, uncorrelated with the error term of the problematic regression in the model. Empirically, the 2SLS regression diagnostic tests allow for gaining a sense of the quality of the selected IVs and the existence of a difference in the estimates obtained with the OLS and 2SLS regressions. For transparency purposes, we recommend reporting these diagnostics tests with the 2SLS regression results. Nevertheless, the decision to include a given IV must also satisfy theoretical scrutiny (Podsakoff et al., 2012). Considering that personality traits influence a wide spectrum of variables in psychology, it might sometimes be difficult to demonstrate their exogeneity in a model. Recent findings regarding relatively short-term changes in personality traits as a result of clinical interventions also emphasize the necessity to capture the exogenous part of the variance in personality traits (Roberts et al., 2017). We acknowledge that our illustration might be limited in this respect.

Interestingly, in psychology, experiments often consider one or several mediating and dependent variables concurrently (Bullock et al., 2010; Judd and Kenny, 2010). Given that mediation analyses also expect the mediator to be unrelated to the error term, endogeneity also threatens these cases. A manipulation of postulated mediators is therefore desirable (Spencer et al., 2005). Nevertheless, when this is difficult, experimentally manipulated independent variables can potentially be used as instruments to recover a consistent estimate (Gennetian et al., 2008), provided that a *full* mediation is hypothesized and that diagnostics confirm its plausibility.

Instrumental variables are, however, not a cure to all illnesses and come with their own limits. If the correlation between instruments and the endogenous explanatory variable is weak, the standard errors of the estimate of interest can be large; the model then necessitates a very large sample (Angrist and Krueger, 1991). Stock and Wright (2000) have formalized the problem and provided critical values that can guide researchers in deciding whether their instruments are weak. Moreover, even a small correlation between the IVs and the error term could create a problem in obtaining consistent estimates and lead to 2SLS estimates that are worse than those of OLS regression (Wooldridge, 2009). Accordingly, it is strongly recommended to have more IVs than endogenous explanatory variables. This allows for an overidentification test, examining whether the instruments are indeed uncorrelated with the error term. Because of these potential limitations, if the Hausman test offers no indication of endogeneity at all, it is usually recommended to base one’s conclusion on the OLS estimates (Wooldridge, 2009), highlighting the importance of reporting the results obtained with OLS regressions as well.

### Time-Related Issues and the Study of Intraindividual Changes

In discussing and positioning IV estimation in the broader landscape of existing design and statistical methods, two related issues regarding time seem relevant. The first refers to the delay between the measurement of cause and effect and its implication for the interpretation of the IV regression results. The second concerns the use of IV in panel designs (i.e., more or less intensively repeated measurements of presumed causes and effects).

The delay created between the manipulation or measurement of a presumed cause and its effect can be of critical importance to observe a relationship (Spector and Meier, 2014). At times, this delay must concur with the time necessary for the putative cause to exert its influence on the targeted outcome. It cannot be shorter, nor much longer, than the necessary time for the explanatory process to unfold; otherwise, the observable effect may have vanished. This is the case if the process underlying the cause-effect relationship is expected to be transient and/or reversible within individuals (Hanges and Wang, 2012). If the interval is not well chosen, IV estimation provides no insurance to observe the effect. At other times, the interval between the measurements of cause and effect matters less because the underlying mechanism is instead expected to be constant. This is the case if the explanatory process is argued to be constituent of individuals and stable over time and if variations are expected *between* individuals. In these situations, temporal precedence can be seen as a way to prevent bias that may arise from a concomitant measurement (Podsakoff et al., 2012).

Our illustration lies in the second category, and this is visible in the similar conclusions we draw with different delays (cross-sectional vs. lagged measurement). In our sample, the basic affective tendencies tied to the personality traits of neuroticism and extraversion are responsible for differences in job satisfaction between individuals, which also translates into differences in career adaptability. Job satisfaction is not *the cause* of career adaptability but is one important element of a broader constellation of factors that, if collectively present, are likely to contribute to career adaptability. Hence, the effect of job satisfaction on career adaptability can be described as an *inus* condition, an “insufficient but non-redundant part of an unnecessary but sufficient condition” to create the outcome (Mackie, 1974, p. 62; Shadish et al., 2002).

Second, endogeneity threats also affect longitudinal designs and statistical frameworks for the analysis of intraindividual changes. For the purpose of illustration, we adopted an interindividual perspective using cross-sectional and lagged data; such designs and perspectives are still prevalent in psychology. However, interindividual difference frameworks fundamentally differ from those focused on intraindividual changes in the level at which the theoretical explanation for the relationships among variables is situated (Hamaker, 2012). Each framework presents different advantages and shortcomings (for a discussion on interindividual frameworks, see, e.g., Spector, 2019). With respect to intraindividual changes, Hamaker et al. (2015) demonstrated that contrary to widespread beliefs, cross-lagged panel models are biased by constant, time-invariant confounding variables. Therefore, frameworks allowing for models combining inter- and intraindividual effects are desirable. Recent developments in the analysis of longitudinal designs offer an estimation of additional parameters beyond autoregressive and cross-lagged relationships. For example, dynamic structural equations models allow for the inclusion of trends, cycles, variability in residual variances, as well as covariation and interindividual differences in some of these parameters when measurement points are numerous (Zhou et al., 2019). General cross-lagged panels allows for the influence of interindividual differences and time-specific impulses (i.e., residuals; Zyphur et al., 2019).

These frameworks permit elaborate considerations of time and the temporal asymmetry between causes and effects, as they explicitly model some sources of endogeneity (e.g., a simultaneous reverse relationship). However, it is still to be examined whether and how omitted variables bias their results. For example, when individuals share a common environment (e.g., work unit or informal social network), unit-level fixed effects should not be ignored. Events influencing both the cause and effect of interest or some time-specific impulses are another type of omitted variable: unexpected individual or collective career shocks (e.g., the departure of one’s mentor or an ethical scandal breaking out within the organization; Seibert et al., 2013; Akkermans et al., 2018) might subtly affect changes in both job satisfaction and career adaptability, perhaps even with different terms and delays. We see IV estimation and advanced analyses of change as complementary techniques with opportunities for integration. Some statistical packages such as Stata offer commands (i.e., *xtivreg* and its options) to implement IV estimation in the context of panel designs (Baltagi, 2008). Researchers interested in dynamics surrounding discrete events can also refer to regression discontinuity designs for an alternative (Imbens and Lemieux, 2008).

## Conclusion

Instrumental variable estimations such as 2SLS regression offer a readily available and viable alternative for obtaining a consistent estimation of the effect of an endogenous regressor on its outcome. This is the case especially when experimental manipulations are difficult to implement, which we illustrated using personality traits as IVs to control for endogeneity in the career adaptability-job satisfaction relationship. Our results suggested that affective dispositions rooted in personality might indirectly account for the individual differences in career adaptability through people’s satisfaction with their job. We hope to have offered an easy-to-understand illustration of the opportunities and challenges of IV estimation.

## Data Availability

All datasets analyzed for this study are included in the manuscript in the form of a correlation table. Some central results reported in the manuscript can be reproduced with codes available in the Supplementary Material.

## Ethics Statement

An ethics approval was not required for the study per the guidelines of the University of Lausanne, and Swiss laws. Informed agreement was ensured early January, for each wave of the data collection. Participants received an information letter outlining the broad research goals, the relevance of the overall project, its institutional funding, and the policy regarding confidentiality as well as detailed information on the sampling procedure. They were also informed of the different parts of the project, the available formats to participate, and the reward (i.e., 20 Swiss francs coupon). Participation was voluntary and respondents could stop completing the research protocol at any time, without further explanation. Because participation was voluntary, we took participants’ responses to the various questionnaires as indication of consent.

## Author Contributions

GB, SR, AB, and JR developed the study concept. GB carried out the data analyses and refined them with SR. GB, SR, AB, and JR interpreted the results. GB drafted the manuscript, with individual parts also written by SR, AB, and JR. GB provided critical revisions by integrating feedback given by SR, AB, and JR. All authors approved the final version of the manuscript.

## Conflict of Interest Statement

The authors declare that the research was conducted in the absence of any commercial or financial relationships that could be construed as a potential conflict of interest.
