# Supplementary material for: Illustrating Instrumental Variable Regressions Using the Career Adaptability – Job Satisfaction Relationship
Source: Front Psychol. 2019 Jun 28;10:1481. doi: 10.3389/fpsyg.2019.01481 (PMC6611186; doi:10.3389/fpsyg.2019.01481)
Supplement: Supplementary file 1 [file Table_1.DOCX]

**Supplementary Material**

**Measurement Model of Personality**

In a replication of Marsh and colleagues (2010) analyses, we applied confirmatory factor analyses (CFA) and exploratory structural equation modeling (ESEM) to responses to the 60-item NEO Five-Factor Inventory Revised. We specified two measurement models with CFA and two models with ESEM, one with and another without the set of 57 a priori correlated uniquenesses (CUs) between items stemming from the same facet of the original NEO-PI-R (McCrae & Costa, 2004) reported by Marsh and colleagues (2010) in their supplementary material.

The pattern of results displayed in Table A1 is consistent with those reported by Marsh and colleagues (2010), albeit weaker. The CFAs revealed a poor fit, and accounting for a priori CUs improved the fit indices in magnitudes similar to those obtained by these researchers. Also consistent, the ESEM solutions fit the data much better than the CFAs. Still, keeping in mind the necessary caution to transpose cutoff values developed in CFA frameworks to ESEM, the CFI and TLI fell short of usual standards of adequate fit, while the RMSEA was very good. In an exploratory way, we therefore specified a third ESEM measurement model to which we added all 28 CUs between items of the same domain suggested by modification indices (see list below). Here, the CFI was acceptable, the RMSEA very good, and the TLI unsatisfactory.

Overall, in light of past research on the measurement model of personality, these results did not identify unknown pattern, nor raise particular doubt regarding the validity of our assessment of personality, even though they were not entirely satisfying and the last model was exploratory and admittedly over fitted. Rather, we believe they demonstrate the promising potential represented by ESEM for the inclusion of personality in latent variable modeling and for the continued refinement of personality inventories. One might speculate that our somewhat weaker results could be explained by our more heterogeneous sample than the sample of Marsh and colleagues (2010). While they surveyed upper secondary students, our sample was composed of adults from the general working aged population. Moreover, we had German and French-speaking respondents. Considering ESEM is still not widespread, further validation efforts and invariance testing of personality within this statistical framework is necessary. This objective however goes well beyond the scope of this article.

**Additional CUs Based on Modification Indices for Items of the Same Domain**

**Neuroticism**

neo11 WITH neo21; neo16r WITH neo31r; neo21 WITH neo51; neo31r WITH neo46r; neo51 WITH neo56;

**Extraversion**

neo2 WITH neo12r neo17 neo57r; neo12r WITH neo47 neo57r; neo17 WITH neo57r;

neo27r WITH neo57r;

**Conscientiousness**

neo5 WITH neo10; neo10 WITH neo45r; neo20 WITH neo50 neo60; neo10 WITH neo55r;

**Agreeableness**

neo4 WITH neo54r; neo9r WITH neo14r neo59r; neo29 WITH neo34; neo39r WITH neo44r;

**Openness to experience**

neo3 WITH neo28r neo53; neo8 WITH neo33r; neo13 WITH neo48r; neo18r WITH neo53; neo33r WITH neo38;

| Table A1  *Goodness-of-Fit Statistics of Confirmatory Factor Analyses and Exploratory Structural Equation Models of the Five Factor Model of Personality* | | | | | | |
| --- | --- | --- | --- | --- | --- | --- |
| Model and description | χ^2^ | *df* | CFI | TLI | NFParm | RMSEA |
| Marsh et al. (2010) |  |  |  |  |  |  |
| CFA – no CUs | 15,488 | 1700 | .685 | .672 | 190 | .049 |
| CFA – a priori CUs | 12,567 | 1643 | .750 | .731 | 247 | .044 |
| ESEM – no CUs | 8,013 | 1480 | .851 | .821 | 410 | .036 |
| ESEM – a priori CUs | 5,201 | 1423 | .914 | .893 | 467 | .028 |
| This Study |  |  |  |  |  |  |
| CFA – no CUs | 6,028.03 | 1700 | .608 | .592 | 190 | .055 |
| CFA – a priori CUs | 5,110.84 | 1643 | .686 | .662 | 247 | .050 |
| ESEM – no CUs | 3,607.57 | 1480 | .807 | .770 | 410 | .041 |
| ESEM – a priori CUs | 2,976.72 | 1423 | .859 | .825 | 467 | .036 |
| ESEM – a priori CUs + 28 within domain CUs | 2,494.30 | 1395 | .900 | .874 | 495 | .031 |
| *Note*. *df* = degrees of freedom; CFI = comparative fit index; TLI = Tucker–Lewis index; NFParm = number of free parameters; RMSEA = root mean square error of approximation; SRMR = standardized root mean square residual ; ESEM = exploratory structural equation modeling; a priori CUs = a priori correlated uniquenesses (based on the facets of the original instrument). | | | | | | |

**Stata Syntax – IV Regression Model Depicted in Figure 1a.**

** Version info: version 12

**To install the *ivreg2* command:

*findit ivreg2

**Model:

**Endogeneous regressor is in the parentheses before =, instrumental variables after it:

ivreg2 Jobsat4 (Caas3 = Consc Open) Neuro Extra Agrea Gender Age, first

ivendog

**To request help on commands and their possible options:

*help ivreg2

*help ivendog

***Note*. Lines starting with * are not read in Stata. ** precede comments, * precedes optional commands.

**Syntax for R package “lavaan” – IV Regression Model Depicted in Figure 1a.**

## Version info: lavaan 0.6-3; psych 1.8.12

## To install the R package “lavaan”:

# install.packages(c("psych", "lavaan"))

## Model:

library(lavaan)

model <- '

## First stage of the model:

Caas3 ~ Gender + Age + Neuro + Extra + Agrea + Consc + Open

## Second stage of the model; instrumental variables are constrained to 0:

Jobsat4 ~ Caas3 + Gender + Age + Neuro + Extra + Agrea + 0*Consc + 0*Open

## Endogeneity: Correlation between error terms of 1st and 2nd stage equations:

Jobsat4 ~~ Caas3

'

model.fit <- sem(model, estimator = "ML", data = data)

summary(model.fit, fit.measures = T, standardized = T, rsquare = T)

## To request standardized results:

#standardizedSolution(model.fit)

##*Note*. Lines starting with # are not read in R. ## precede comments, # precedes optional commands. As an alternative, the package “systemfit” by Henningsen and Hamann (2007) allows to fit linear structural equations using 2SLS estimation method.

**Mplus Syntax – IV Regression Model Depicted in Figure 1a.**

!! Version info: Mplus 7.1

MODEL:

!! First stage of the model:

Caas3 ON Gender Age Neuro Extra Agrea Consc Open;

!! Second stage of the model; instrumental variables are constrained to 0:

Jobsat4 ON Caas3 Gender Age Neuro Extra Agrea Consc@0 Open@0;

!! Endogeneity: Correlation between error terms of 1st and 2nd stage equations:

Jobsat4 WITH Caas3;

!! To request standardized results:

!OUTPUT: STDYX;

!!*Note*. Lines starting with ! are not read in Mplus. !! precede comments, ! precedes an optional command.

**Syntax for SPSS – IV Regression Model Depicted in Figure 1a.**

** Version info: version 25

** Model:

** The endogeneous regressor is included only after the WITH, instrumental variables only ** after INSTRUMENTS, and control variables after WITH and INSTRUMENTS:

TSET MXNEWVAR=2.

2SLS Jobsat4 WITH Caas3 Gender Age Neuro Extra Agrea

/INSTRUMENTS Gender Age Neuro Extra Agrea Consc Open

/CONSTANT.

***Note*. Lines starting with a * are not read in SPSS. ** precede comments.

**References**

[Baum, C. F.,](https://www.sciencedirect.com/science/article/pii/S1048984310001475#bbb0095) Schaffer, M. E., & Stillman, S. (2007). Enhanced routines for instrumental variables/generalized method of moments estimation and testing. *The Stata Journal, 7*(4), 465-506.

Henningsen, A., & Hamann, J. D. (2007). systemfit: A package for estimating systems of simultaneous equations in R. *Journal of Statistical Software, 23*(4), 1-40.

Marsh, H. W., Lüdkte, O., Muthén, B., Asparouhov, T., Morin, A. J. S., Trautwein, U., & Nagengast, B. (2010). A new look at the Big Five factor structure through exploratory structural equation modeling. *Psychological Assessment, 22,* 471–491. doi:10.1037/a0019227

Rosseel, Y. (2012). lavaan: An R package for structural equation modeling. *Journal of Statistical Software, 48*(2), 1-36.
